# A new voice for the Western Pacific region

**DOI:** 10.1016/j.lanwpc.2020.100015

**Published:** 2020-09-04

As of Aug 11, 2020, the number of confirmed cases of COVID-19 has reached over 197 million globally, with more than 0•7 million deaths. Parallel with the spread of this viral pandemic is the so-called infodemic, defined by WHO as “an overabundance of information—some accurate and some not—that makes it hard for people to find trustworthy sources and reliable guidance when they need it.” The infodemic is fuelled by the misuse of social media and the internet; the misinformation and conspiracy theories that it carries undermine public trust, amplify fear, and ultimately damage efforts to control the pandemic both globally and locally.

In its first infodemiology conference in June and July, 2020, WHO gathered experts from various disciplines, including epidemiology, public health, data science, social science, and media studies. They aimed to discuss, understand, and find feasible measures to confront disinformation. Trustworthy sources of information are needed more than ever, and peer-reviewed scientific publications play a crucial part in providing a validated evidence base. To date, more than 38 000 COVID-19-related articles have been indexed in PubMed. Even though the rapid publication speed during the past 7 months has raised some concerns about the quality and potential bias and limitations of some scientific publications, on the whole, valuable knowledge has been gained from basic research on the virology and pathogenesis of COVID-19, from clinical observations on epidemiology and viral transmission, and from clinical trials on potential treatment and management modalities for patients with COVID-19.

The Western Pacific region is home to more than a quarter of the world's population, with vast differences in the quality and availability of health-care services in each country and area. Not only does the COVID-19 pandemic affect each country differently, but it also disparately affects each community within a country depending on socioeconomic status. Coping with COVID-19 is already overwhelming for some low-resource countries and areas where the pandemic is often compounded by an existing high prevalence of underlying health conditions such as diabetes and heart disease.

To this end, *The Lancet* Group launched *The Lancet Regional Health – Western Pacific*, the first journal in a suite of open-access general medical journals for the publication of high-quality, evidence-based research from six WHO-defined regions. Since May, 2020, when the submission site was opened, the journal has received high-quality research articles, commentaries, letters, reviews, and health policy papers from many countries and areas in the region, including Australia, China, Fiji, Indonesia, Japan, Laos, Malaysia, New Zealand, Papua New Guinea, Philippines, Samoa, Singapore, South Korea, and Vietnam. This inaugural issue of the journal highlights a selection of regional research and commentary, adding reliable evidence and raising awareness of health issues related but not limited to COVID-19 in Western Pacific countries.

Hiroaki Miyata and colleagues used a social networking service messaging application called COOPERA to monitor COVID-19-related symptoms in Fukuoka Prefecture, Japan. The authors showed that social media platforms can be a useful tool to monitor local outbreaks during the pandemic, as opposed to spreading misinformation. Another modelling study led by Alex Cook and colleagues brought new evidence that the lockdown and gradual exit strategies in Singapore could avoid a resurgence of COVID-19 cases. In a comment Aryati Yashadhana and colleagues pointed out how health and socioeconomic inequities might increase the risk of COVID-19 in Indigenous Australians, and appealed for the implementation of appropriate approaches in this susceptible group during the pandemic.

Besides COVID-19-related articles, Brian Chung and colleagues suggest that rapid whole-exome sequencing could deliver clinically useful data in a timely and cost-effective manner in a paediatric and adolescent population in Hong Kong, who might harbour rare genetic disorders. Georgina Phillips and colleagues did a three-phase expert consensus process in Pacific island countries and territories, and provided crucial evidence for the development of emergency care with defined priorities and standards specific for this region.

*The Lancet Regional Health – Western Pacific* aims to publish high-quality, reliable evidence on clinical practice and health care in the region. The journal aspires to be a leading and open forum for constructive dialogue and discussions among regional health practitioners, communities, and policy makers. Through collaboration, it is hoped that universal, high-quality, and assessible health care can be achieved throughout the Western Pacific region.

*The Lancet Regional Health – Western Pacific*

